# Effect of cord blood double collection method on cord blood hematopoietic stem cell transplantation-related indices and blood gas analysis

**DOI:** 10.1097/MD.0000000000036227

**Published:** 2023-11-24

**Authors:** CongYing Shi, WenYu Rao, WanJun Huang, TianBao Ma, Wei Wei

**Affiliations:** a Institution of Guangdong Cord Blood Bank, Guangdong Women and Children Hospital, Guangzhou, Guangdong, China; b Department of Experimental Center, Guangzhou Municipality Tianhe Nuoya Bio-engineering CO., Ltd, Guangzhou, Guangdong, China; c Department of Obstetrics, Nanhai Fourth People’s Hospital, Foshan, Guangdong, China.

**Keywords:** base excess, CD34+, CFU-GM, pH, TNC

## Abstract

**Background:**

Umbilical cord blood has been widely used in clinical transplantation. Blood gas analysis of umbilical cord blood is routinely used to evaluate neonatal asphyxia. This study aimed to evaluate an improved umbilical cord blood collection method that does not affect the results of umbilical cord blood gas analysis and hematopoietic stem cell transplantation-related indices.

**Methods:**

Three hundred pregnant women were recruited between December 2019 and August 2022. In total, 270 umbilical cord blood samples were included and randomly divided into 3 groups. Group A was defined as the group in which both umbilical cord blood samples for hematopoietic stem cell transplantation and blood gas analysis were collected. Group B was defined as the group from which umbilical cord blood was collected for hematopoietic stem cell transplantation. Group C was defined as that wherein umbilical cord blood was collected only for blood gas analysis. Hematopoietic stem cell transplantation-related indices were detected in groups A and B, and blood gas analysis was performed in groups A and C.

**Results:**

Hematopoietic stem cell transplantation-related indices were not significantly different between groups A and B. The pH, base excess, and lactic acid values were not significantly different between groups A and C.

**Conclusion:**

The cord blood double collection method would not affect the results of umbilical cord blood gas analysis and hematopoietic stem cell transplantation-related indices. It is suitable for cord blood collection when preparing for hematopoietic stem cell transplantation and blood gas analysis.

## 1. Introduction

Most neonates actively experience breathing air after birth; however, 10% require active resuscitation for active breathing and 1% require extensive care. The inability of neonates to breathe results in hypoxia. If timely treatment is not provided, hypoxia could lead to hypoxic ischemic encephalopathy (HIE) or neonatal death.^[1]^ Therefore, each newborn is given an Apgar score, which is often used by nurses as the main indicator for diagnosing neonatal asphyxia in postpartum mothers. The Apgar score system is a rapid method for assessing the clinical status of newborns 1 and 5 minutes after birth.^[2]^ However, this is not an ideal method for assessing outcomes.^[3]^ Most patients with HIE have normal Apgar scores at birth. Umbilical cord arterial blood gas (UC-ABG) analysis is an objective and effective auxiliary test for assessing the oxygenation and metabolic status of neonates at birth, in which pH could be used as a predictor of adverse neonatal outcomes. The UC-ABG analysis is a practical technology for screening high-risk newborns.^[4]^ UC-ABG obtained at the time of delivery allows for the assessment of fetal acid-base status. A meta-analysis showed that fetal acidemia at delivery is associated with adverse neonatal and long-term outcomes.^[5]^ The American College of Obstetricians and Gynecologists and the American Academy of Pediatrics consider it an essential indicator for the diagnosis of neonatal asphyxia.^[6]^ Therefore, collection of umbilical cord blood (UCB) for blood gas analysis is necessary and frequently performed.

UCB is rich in various stem cells and nutrients, including hematopoietic stem cells, mesenchymal stem cells, endothelial progenitor cells, unrestricted somatic cells, and very small embryonic/ectoderm-like stem cells.^[7]^ The ease of collection, immunological naivety, physical storage,^[8]^ and potential as a new source of hematopoietic stem cell transplantation are the advantages of UCB.^[9]^ In an extensive clinical application and in-depth study of UCB by researchers, it was reported that cord blood is not only used to treat blood system diseases but also widely used to treat various immune deficiencies, metabolic diseases, and neurological diseases and used in regenerative medicine.^[10]^ At the UCB public bank, cord blood is available for patients in need of hematopoietic stem cell transplantation nationwide. The requirements for UCB hematopoietic stem cell transplantation-related indicators, including UCB volume, total mononuclear cell count (TNC), CD34^+^ cell content, and colony-forming unit granulocytes and macrophages (CFU-GM), are high and standardized.^[11]^ UCB collection for blood gas analysis might have an impact on the indices related to cord blood hematopoietic stem cell transplantation.

We hypothesized that umbilical vein blood could be used for both blood gas analysis and hematopoietic stem cell preparation This study is the first to investigate the feasibility of umbilical vein blood collection for both blood gas analysis and preparation of umbilical blood hematopoietic stem cells. Providing a new feasible scheme for collecting cord blood for blood gas analysis without affecting the indices related to cord blood hematopoietic stem cell transplantation.

## 2. Materials and methods

### 2.1. Study design

A total of 300 participants were selected from the UCB collected from a grade III public hospital between August 2018 and December 2021 according to the inclusion criteria. A previous trial found that the mean ± standard deviation of UCB volume was 74.73 ± 28.02. We hypothesized the mean of UCB volume were 70 and 74 as group A and B, standard deviation was 10. And calculated that a sample size of 78 patients per group would provide 80% power at Type I error rate value of 10% to detect the difference in UCB volume between 2 groups. To account for possible protocol violations, we enrolled a total of 100 participants each group. The inclusion criteria were as follows: maternal age 18 to 40 years, 37 to 42 weeks of gestation, vaginal delivery, singleton pregnancy, consented to donating or depositing cord blood, and availability of complete maternal information records. The exclusion criteria included non-vaginal delivery and partially missing test data. The women were randomly divided into 3 groups of 100 each by random selection method. Group A was defined as the group in which UCB samples for hematopoietic stem cell transplantation and blood gas analysis were collected. Group B was defined as that wherein UCB was collected for hematopoietic stem cell transplantation. Group C was defined as that wherein UCB was collected only for blood gas analysis. Hematopoietic stem cell transplantation-related indices were detected in groups A and B, and blood gas analysis was performed in groups A and C. The chief head nurse of the hospital generated the random allocation sequence, enrolled participants, and assigned participants to interventions. The analyst was unaware of the grouping.

All cord blood samples were sent to the Guangdong Cord Blood Hematopoietic Stem Cell Bank (GDCBB) for UCB preparation, testing, and storage. All UCB samples were prepared in accordance with the requirements of relevant Chinese laws and regulations.^[9]^ Trained nurses, obstetricians, and midwives collected the UCB samples from mothers who provided informed consent. All participants tested negative for hepatitis B surface antigen, hepatitis C antibody, syphilis antibody, and acquired immune deficiency syndrome (AIDS) antibody, and none had thalassemia and G-6-phosphate glucose dehydrogenase deficiency.

### 2.2. Methods and data collection

**Figure 1.**
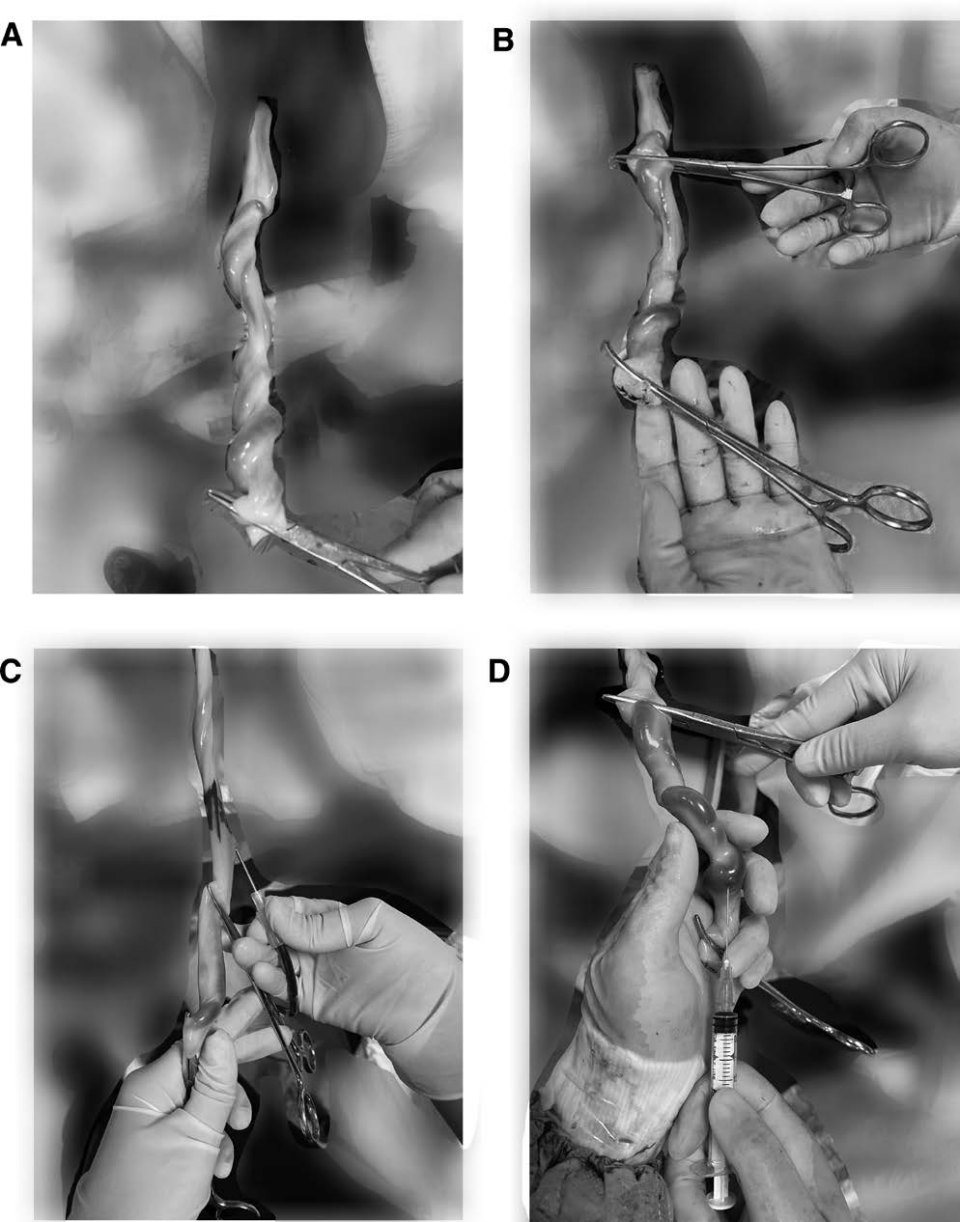
UCB double collection method. (A) Clamping the umbilical cord with the first hemostatic clamp and cutting off. (B) Clamping 3–4 cm away from the first hemostatic clamp by another hemostatic clamp. (C) Collecting UCB for separating hematopoietic stem cells. (D) Collecting UCB for blood gas analysis. UCB = umbilical cord blood.

In group A, the neonate was successfully delivered. Before the newborn infant uttered the first cry, the umbilical cord was clamped using the first hemostatic clamp and cut off. The umbilical cord was clamped 3 to 4 cm away from the first hemostatic clamp using another hemostatic clamp, and sufficient umbilical blood was reserved for blood gas analysis. The UCB for separating hematopoietic stem cells was first collected. The cord blood collection procedure was as follows: the UCB collection site was 15 cm from the placenta on the umbilical cord, and the umbilical cord collection site was disinfected with 75% alcohol and then fixed with sterile gauze to prevent slipping. When performing umbilical vein puncture, the tip of the blood collection needle was inclined downward or laterally. The needle moved along the vascular wall 1 to 2 cm after the blood was observed. When the UCB flowed into the blood bag, the bag was gently shaken to allow the anticoagulant to fully mix. The UCB was collected until the cord turned gray and UCB collection was completed. Another needle was used to collect the UCB for blood gas analysis. The procedure was as follows: 12,500 units of heparin was dissolved in 0.9 % saline 100m, and after draining 1 ml of the wetting injector with a 2 ml syringe, the liquid with heparin was pushed out. Approximately 2–4 mL of umbilical cord vein blood was collected from the umbilical cord between the first and second forceps. After collection, the samples were immediately sealed and sent for blood gas analyses. The UCB collection method is shown in Figure 1.

Group B: Collection of UCB for separating hematopoietic stem cells. The procedure was the same as that in group A.

Group C: Collection of UCB only for detecting blood gas. The procedure was the same as that used for group A.

After collecting the UCB for separating hematopoietic stem cells, it was immediately stored in a refrigerator at 0 to 4°C and transported to the GDCBB within 24 hours.

### 2.3. Materials

Heparin(Changzhou Qianhong, China), UCB collection bags (Shandong Weigao, Weihai, China), 6% hydroxyethyl starch (Hespan, B. BRAUN, Melsungen, Germany), 1% hemolysin, 7-aminoactinomycin D, CD45-fluorescein isothiocyanate monoclonal antibody, CD34-phycoerythrin monoclonal antibody, and IgG1-phycoerythrin monoclonal antibody were purchased from BD Biosciences (New York, USA). Blood gas electrolyte analyzer(BG-800E, Meizhou Kangligao), Medical cryogenic centrifuge (RC 3BP+, Thermo Scientific), automatic blood analyzer (XE-5000, Sysmex), flow cytometer (FACSCalibur, BD), inverted microscope (CK40 and CKX41, OLYMPUS), and Automatic Microbial Detection System (VersaTREK, ThermoFisher) were used in the study.

### 2.4. Method of UCB hematopoietic stem cell separation and detection

All UCB hematopoietic stem cell separation and cryopreservation were performed in GDCBB. Hematopoietic stem cell transplantation-related indices included TNC, mononuclear cell count (MNC), detection of hepatitis B antigen, hepatitis C antibody, syphilis antibody, AIDS antibody, cytomegalovirus antibody, and bacteria/mold contamination, CFU-GM count, and CD34^+^ cell count.

### 2.5. Statistics

SPSS software (version 18.0) was used for data analysis. The measurement data that conformed to the normal distribution are expressed as the mean ± standard deviation (x̅ ± s), and the differences between the 2 groups were compared using an independent sample t-test. Differences among the 3 groups were compared using one-way analysis of variance. Count data, expressed as percentages (%), were analyzed using the chi-squared (χ^2^) test. *P* < .05 was considered statistically significant.

### 2.6. Ethics approval and informed consent

The ethics committee of Guangdong Cord Blood Bank approved this study (No. IRB-2022004). All procedures followed were in accordance with the ethical standards of the responsible committee on human experimentation (institutional and national) and with the Helsinki Declaration of 1975, as revised most recently in 2013. All participants had provided their written informed consent to this study. The study data did not involve information that could identify individual participants.

## 3. Results

### 3.1. Participant recruitment and general information

**Figure 2.**
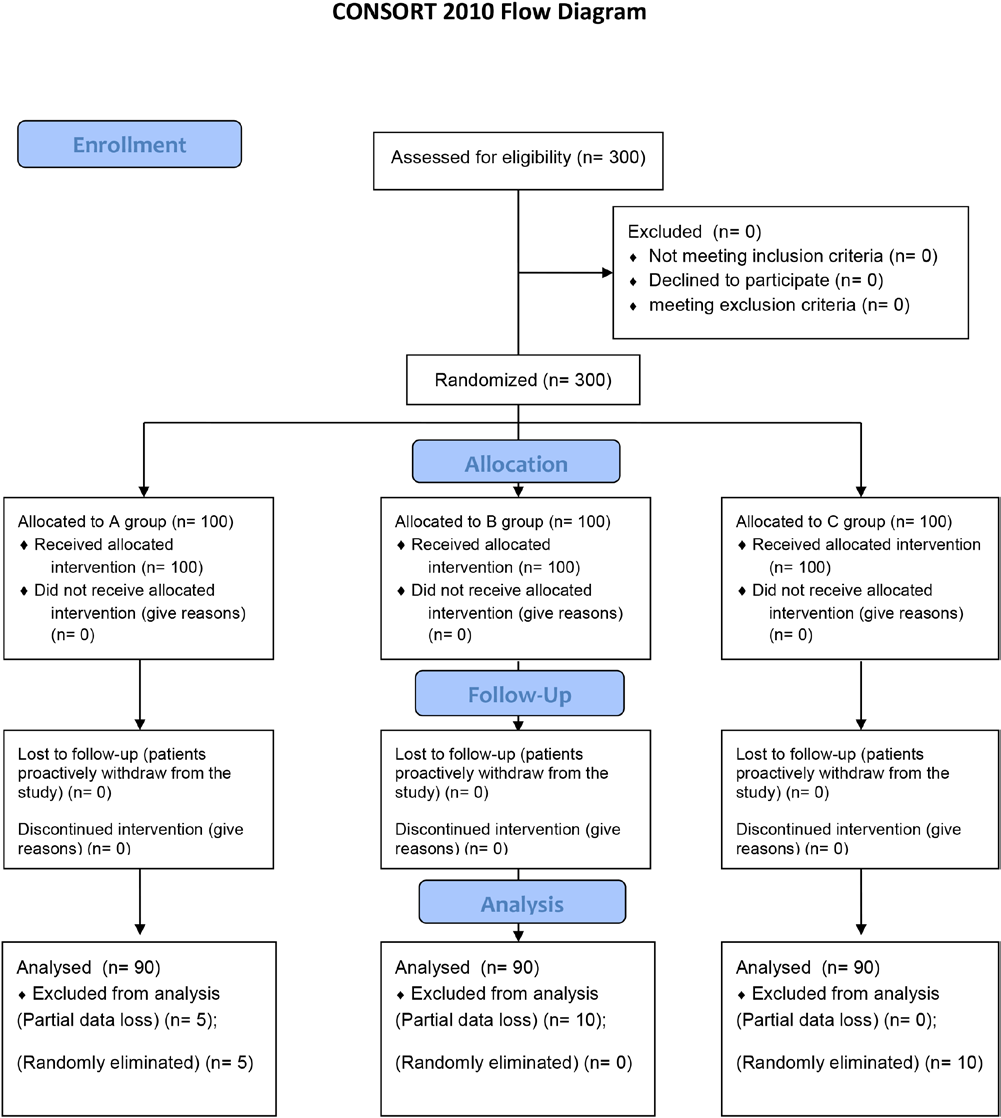
CONSORT Flow Diagram.

After screening the data according to the exclusion criteria, 90 participants were randomly selected from each group for the analysis. In total, 270 UCB samples were collected. A CONSORT flow diagram is shown in Figure 2. There was no significant difference in either the general maternal information (*P* > .05) (Table 1) or pairwise comparisons between the 3 groups (*P* > .05).

**Table 1:** General maternal information of the participants.

Group	Age (year)	Gestational week (week)	Delivery times, N (%)				Delivery method, N (%)		Sex of newborn, N (%)	
			One	Two	Three	Four	Natural labor	Forceps delivery	Male	Female
A	29.96 ± 3.80	39.25 ± 1.11	38 (42.2)	40 (44.4)	10 (11.1)	2 (2.2)	90 (100)	0 (0)	45 (50)	45 (50)
B	29.47 ± 4.33	39.31 ± 1.00	34 (37.8)	53 (58.9)	3 (3.3)	0 (0)	87 (96.7)	3 (3.3)	39 (43.3)	51 (56.7)
C	28.62 ± 4.33	39.24 ± 1.12	34 (37.8)	43 (47.8)	11 (12.2)	2 (2.2)	89 (98.9)	1 (1.1)	50 (55.6)	40 (44.4)
F	2.365	0.116	0.863				1.780		1.347	
*P*	0.096	0.891	0.423				0.171		0.262	

### 3.2. Analysis of hematopoietic stem cell transplantation-related indices in groups A and B

The UCB hematopoietic stem cell transplantation-related indices of the 2 groups are shown in Table 2. Hepatitis B antigen, hepatitis C antibody, syphilis antibody, AIDS antibody, cytomegalovirus antibody, and bacteria/mold contamination detection were negative in both groups. The UCB volume, TNC, MNC, CFU-GM, CD34^+^ content, and blood type were not significantly different between the 2 groups (*P* > .05).

**Table 2:** UCB hematopoietic stem cell transplantation-related indices of groups A and B.

Group	UCB volume (mL)	TNC (×10^8^)	MNC (×10^8^)	CFU-GM (/10^5^ cell)	CD34^+^ content (%)	Blood type, n (%)			
						A	B	O	AB
A	71.37 ± 25.52	10.21 ± 5.25	3.58 ± 1.78	9.36 ± 6.59	35.62 ± 20.30	24 (27.0)	23 (25.8)	11 (12.4)	31 (34.8)
B	74.73 ± 28.02	9.83 ± 5.03	3.81 ± 1.89	9.42 ± 7.39	37.04 ± 28.29	21 (24.4)	22 (25.6)	7 (8.1)	36 (41.9)
t	0.843	-0.484	0.835	0.060	-0.168				
χ^2^						1.433			
*P*	0.401	0.629	0.405	0.952	0.866	0.698			

CFU-GM = colony-forming unit granulocytes and macrophages, MNC = mononuclear cell count, TNC = total mononuclear cell count, UCB = umbilical cord blood.

### 3.3. Blood gas analysis of groups A and C

The UCB blood gas analysis data for the 2 groups are shown in Table 3. The pH, base excess (BE), and lactic acid (Lac) values of the 2 groups were not significantly different (*P* > .05). In contrast, the partial pressure of oxygen (PaO_2_) values of the 2 groups were significantly different (*P* < .05).

**Table 3:** UCB blood gas analysis of groups A and C.

Group	pH	BE	PaO_2_	Lac
A	7.34 ± 0.62	–4.22 ± 3.18	21.88 ± 9.63	3.11 ± 0.60
C	7.33 ± 0.65	–3.73 ± 2.24	31.14 ± 11.08	3.10 ± 0.66
t	1.187	–1.172	–5.985	0.131
*P*	.237	.243	.000	.896

BE = base excess, PaO_2_ = partial pressure of oxygen, Lac = lactic acid.

## 4. Discussion

UC-ABG analysis can determine the degree of fetal intrauterine hypoxia, which reflects the acid-base status of the fetus, and is the main method used to evaluate the fetal acid-base status and gas and substance metabolism. The neonatal central nervous system has an extremely poor tolerance to hypoxia, and central nervous system damage in asphyxiated children begins with disturbances in umbilical blood flow and fetal gas exchange. One of the most devastating complications of HIE is cerebral palsy, with children with cerebral palsy having the highest burden on families, and the effects persist throughout life. Timely and accurate diagnosis of asphyxia and correct resuscitation could reduce the incidence of asphyxia, cerebral palsy, and neonatal mortality.^[12]^ A recent study showed that in preterm neonates at < 29 weeks’ gestation, low umbilical cord arterial pH and high BE were associated with a clinically important increase in the post-test probability of mortality.^[13]^ The condition and degree of asphyxia in children can be judged using UC-ABG analysis, which is beneficial for formulating clinical treatment plans. The UC-ABG values are considered more accurate in reflecting the fetal acid-base status than venous blood gas values. However, obtaining umbilical cord arterial blood is difficult. One study has pointed out a strong correlation between the blood gas values of arterial cord blood and venous cord blood and that the blood gas value of venous cord blood can be used as an indicator of neonatal asphyxia.^[14]^ When umbilical artery blood sample is difficult to obtain, umbilical vein blood sample is the second choice for blood gas analysis. From the perspective of cord blood hematopoietic stem cell collection, we hypothesized for the first time that there can be a speedy method for umbilical vein blood collection. The method simultaneously meets the requirements of quality of cord blood hematopoietic stem cell collection and clinical blood gas analysis evaluation. This approach can reduce the workload of doctors in the delivery room, allowing more time to focus on women and newborns.

Our study showed that the pH, BE, and Lac values of venous blood collected using this cord blood double collection method were relatively stable. According to previous studies, pH, BE, and Lac levels are significance predictors of adverse neonatal outcomes.^[15]^ Therefore, we believe that blood gas analysis using this collection method can be used as an indicator to evaluate fetal acidemia and neonatal asphyxia. Although the pH prediction of neonatal outcomes remains imperfect, it is one of the best evaluation tools available, and pH information is important for neonatologists and legal issues.^[16]^ There were significant differences in the PaO_2_ values in this study, but Andres et al pointed out that umbilical artery PaO_2_ also had no obvious clinical utility.^[17]^ In our study, a newborn with 1 minute Apgar score of 7 was diagnosed with mild asphyxia. These symptoms were caused by amniotic fluid turbidity. The pH, BE and the Lac values were 7.33, −4.67, and 3.37, respectively. The Apgar score recovered to 10 points at 5 minutes after treatment, and the subsequent vital characteristics of the newborn were normal. Therefore, it is meaningful to collect umbilical cord venous blood for blood gas analysis to assess the reason of fetal acidemia and asphyxia in neonates and collect hematopoietic stem cells simultaneously.

Some studies have suggested a negative correlation between pH and the concentrations of CD34 + cells and TNCs.^[18]^ The results of the venous and arterial blood tests were inconsistent. A volume of 3 to 4 mL of UCB is needed for the UC-ABG. Improper collection of UCB for UC-ABG might result in more wasted cord blood, which would directly affect the indices of UCB collected for hematopoietic stem cell transplantation.^[19]^ Whether collecting venous UCB for blood gas analysis affects hematopoietic stem cell transplantation-related indices remains unknown. A randomized controlled trial showed that umbilical cord venous blood gas analysis values remained stable for up to 60 minutes even when the cord ends were clamped and placed on a bedside birth tray.^[20,21]^ This conclusion is the key theoretical basis for the method of collecting UCB for blood gas analysis and hematopoietic stem cell analysis.

Unrelated UCB donated to public banks for the common good offers substantial promise as a graft for hematopoietic stem cell transplantation, a source of different cell subsets for cellular therapies,^[22]^ and as a starting material for ex vivo expansion, cell engineering, and cell reprogramming technologies.^[23]^ In people with normal immune function, allogeneic cells are cleared by autoimmune cells through an immune response. In clinical trials of allogeneic mesenchymal stem cells, multiple transfusions of stem cells are usually required to achieve therapeutic effects.^[24,25]^ Patients requiring allogeneic UCB hematopoietic stem cell transplantation generally received some degree of myeloablative therapy, and TNC > 2.5 × 10^7^/Kg and CD34^+^ cells > 1.5 × 10^5^/Kg of UCB are recommended.^[11]^ Therefore, the number of allogeneic stem cells is important for allogeneic hematopoietic stem cell transplantation or allogeneic stem cell infusion therapy. As UCB for blood gas analysis is routinely collected from newborns in China, an improper operation might seriously affect the quality of UCB after collecting part of the UCB for blood gas analysis; this might lead to the hematopoietic stem cell transplantation-related indices of UCB not matching the clinical requirements. Substandard UCB would then be scrapped, which would be quite a waste. Our study showed that the use of the improved UCB collection method effectively ensured that the impact on the relevant indices of hematopoietic stem cell transplantation was minimized based on the need to draw UCB for blood gas analysis. The hematopoietic stem cell transplantation-related indices of UCB were not significantly different between groups A and B (*P* > .05).

This study had some limitations. First, the data in this study were only from the UCB collected from vaginal deliveries, and subsequent studies can further include UCB data from cesarean deliveries. There was limited data of neonates diagnosed with asphyxia in this study, and the results only showed that this method is applicable to normal neonates. Subsequent studies can be carried out on neonates diagnosed with neonatal asphyxia, but UCB collection should be based on the safety and health of neonates.

In conclusion, the UCB double collection method not only meets the requirements of UCB blood gas analysis but also minimizes the impact on hematopoietic stem cell transplantation-related indices of UCB. This finding deserves attention in the field of UCB collection.

## Acknowledgments

The authors would like to express their deepest appreciation to all colleagues of the Obstetrics Department of Nanhai Fourth People Hospital for their help in collecting data. We would also like to thank Editage (www.editage.cn) for English language editing. This research received no specific grant from any funding agency in the public, commercial, or not-for-profit sectors.

## Author contributions

**Conceptualization:** Shi Congying, Wei Wei.

**Data curation:** WenYu Rao, TianBao Ma.

**Formal analysis:** Shi Congying, WanJun Huang, TianBao Ma.

**Investigation:** WenYu Rao, WanJun Huang, TianBao Ma.

**Methodology:** Shi Congying, WenYu Rao, WanJun Huang, Wei Wei.

**Project administration:** Shi Congying, WenYu Rao, Wei Wei.

**Software:** Shi Congying.

**Supervision:** Shi Congying, Wei Wei.

**Validation:** Shi Congying, WenYu Rao, WanJun Huang, TianBao Ma, Wei Wei.

**Visualization:** Shi Congying.

**Writing – original draft:** Shi Congying, WenYu Rao.

**Writing – review & editing:** Shi Congying, WenYu Rao, WanJun Huang, TianBao Ma, Wei Wei.
